# Tourists’ Health Risk Threats Amid COVID-19 Era: Role of Technology Innovation, Transformation, and Recovery Implications for Sustainable Tourism

**DOI:** 10.3389/fpsyg.2021.769175

**Published:** 2022-04-07

**Authors:** Zhenhuan Li, Dake Wang, Jaffar Abbas, Saad Hassan, Riaqa Mubeen

**Affiliations:** ^1^School of Humanities, Ningbo University of Finance and Economics, Ningbo, China; ^2^School of Media and Communication, Shanghai Jiao Tong University, Shanghai, China; ^3^Antai College of Economics and Management, Shanghai Jiao Tong University, Shanghai, China; ^4^Air University School of Management, Air University, Islamabad, Pakistan; ^5^School of Management, Harbin Institute of Technology, Harbin, China

**Keywords:** COVID-19, psychological reactance, social distance, consumer behavior, employees’ workplace behavior, B2C, tourism recovery strategies

## Abstract

Technology innovation has changed the patterns with its advanced features for travel and tourism industry during the outbreak of COVID-19 pandemic, which massively hit tourism and travel worldwide. The profound adverse effects of the coronavirus disease resulted in a steep decline in the demand for travel and tourism activities worldwide. This study focused on the literature based on travel and tourism in the wake global crisis due to infectious virus. The study aims to review the emerging literature critically to help researchers better understand the situation. It valorizes transformational affordance, tourism, and travel industries impacts posed by the virus COVID-19. The study proposed a research model on reviving the international tourism activities post COVID-19 pandemic to gain sustainable development and recovery. The scholars have debated seeking the best possible ways to predict a sustainable recovery of travel, tourism, and leisure sectors from the devastating consequences of coronavirus COVID-19. In the first phase, the study describes how the current pandemic can become transformational opportunities. It debates the situation and questions related to the emergence of the COVID-19 outbreak. The present research focuses on identifying fundamental values, organizations, and pre-assumptions related to travel and tourism revival and help academia and researchers to a breakthrough in initiating the frontiers based on research and practice. This study aims at exploring the role of technological innovation in the crisis management of COVID-19 tourism impacts, tourists’ behavior, and experiences. The travel and tourism industry’s main stakeholders include tourism demand and organizations that manage tourists’ destinations and policymakers. They have already experienced the stages of responses, recovery, and resetting tourism recovery strategies. The study provides valuable insight into the coronavirus consequences on travel and tourism and practical implications for global tourism and academic research revitalization.

## Introduction

The COVID-19 virus transmits from individuals’ interaction and physical contact, giving rise to mobility bans, travel restriction challenges, and community lockdowns as vaccine availability still remains a global challenge (Rothan and Byrareddy, 2020; Shi et al., 2020; Su et al., 2021a,b). As a result, psychological fear and anxiety emerged that compelled people to avoid social gatherings. Staying at home caused devastating psychological effects on the hospitality, leisure and tourism industries (Gössling et al., 2020; Yin and Ni, 2021). The pandemic has posed challenges on how tourists can cope with the COVID-19’s consequences for travel, hospitality and tourism activities worldwide (Ge et al., 2021; Liu et al., 2021; Mubeen et al., 2021a,b). Travel challenges have gained much attention from academics and researchers of tourism. Scholars argued that business managers and organizations’ owners of the tourism industry are keenly interested in finding alternatives to discover innovative ways to revive tourism activities amid the COVID-19 challenging situation (Duarte Alonso et al., 2020). The literature has shown a steep decline in tourism activities due to the emergence of the pandemic COVID-19. Scholars have paid attention to discover new safe ways to minimize tourists’ psychological stress from the perspective of tourism firms; as less occupancy and limited rooms’ availability, operations in travel and tourism have significantly declined worldwide (Buckley and Westaway, 2020; Park et al., 2020, 2021).

The steep decline in tourism activities has resulted in massive unemployment as service firms laid off employees due to bans and closure of business operations (Kreiner and Ram, 2020; Kaushal and Srivastava, 2021; Tu et al., 2021). Tourism restrictions and travel bans have resulted in mental stress and psychological problems among employees. They have felt the threat of layoff and increasing unemployment ratio that has affected hospitality and tourism firms’ employees performance. Thus, under the circumstances of the COVID-19 pandemic, hospitality and tourism firms’ have experienced substantial economic losses, and employees have seen sadness, uncertainty and anxiety. Thus, employees psychological state, emotions, and behavioral reactions are crucial to face the challenges of the COVID-19 pandemic (Sah et al., 2020). As a result, hospitality and tourism firms’ employees have shown fear of providing physical services for tourists due to the chance that the COVID-19 transmission might be asymptomatic (Aguiar-Quintana et al., 2021). The past literature has shown fear, anxiety and adverse psychological state of employees working in the tourism industry (Vu et al., 2021). Thus, the pandemic COVID-19 emergence has negatively influenced employees performance working in travel, hospitality and tourism firms (Deffenbacher, 1977; Stefan and David, 2013; Frode et al., 2017; Sklett et al., 2018; Narayanamurthy and Tortorella, 2021).

The COVID-19 pandemic resulted in a massive crisis for the travel and tourism industries. The advent of the coronavirus outbreak has significantly affected global social, political, economic, and socio-cultural systems worldwide. Health communications and prevention measures, such as staying at home, mandatory-quarantine campaigns, social distancing, mobility and travel bans, border closure, and community lockdowns, have halted the travel and tourism industry. It is a highly vulnerable sector to numerous risk factors. Still, tourism has become resilient in reviving to the next normal from various epidemics and pandemics, such as earthquakes, MERS, SARS, Ebola, and Zika (Novelli et al., 2018). Thus, the unprecedented settings, nature, and the COVID-19 pandemic impacts demonstrate indication that the crisis is distinct and will have profound long-term transformational and structural changes to the travel, leisure, and tourism industry (Škare et al., 2021). The pandemic posed huge adverse effects because of the global travel restrictions and a sharp slump in tourism demand among tourists and travelers (Verma and Gustafsson, 2020). The coronavirus outbreak has massively affected the tourism industry as many states and territories imposed travel restrictions and social gatherings in attempts to contain the COVID-19 disease spread (Aleta et al., 2020). The travel and tourism industry is one of the most affected industries as this sector has met the hardest hit of the COVID-19 pandemic. The global internal travel and tourism activities showed a 51% drop in revenue, amounting to 2.86 trillion U.S. dollars. Besides, the experts have forecasted the tourism market to recovery rapidly, and it will reach the next normal levels of 2019 by 2023. High-end luxury tourism is vital, and the leisure industry paid particular attention to satisfy the lavish tourists’ travel expectations, as they are high-net-worth travelers. This tourism segment also saw a steeper decline in 2020.

The global internal luxurious tourism saw a 54% decline, which resulted in a slump in revenue and business growth worldwide. According to an estimation, the United Nations World Tourism Organization reported that global tourists and travelers plans might decline by 60–80% in 2020 (Neuburger and Egger, 2020). It would lead to a potential economic loss of 0.9 to 1.20 trillion dollars in the international tourism business. Many tourist places and cities reported an 80–90% decline in planned travel (Bhaskara and Filimonau, 2021). Conflicting and unilateral tourism restrictions on travelers occurred regionally (Kallbekken and Sælen, 2021), and many global tourist attractions, such as amusement parks, sports venues, and museums, were under visitor restrictions (Oum and Wang, 2020). UNWTO specified a 65% decline in international tourists’ arrivals in the first half of 2020 (Miech et al., 2021). Air passengers’ travel reported a drop of more than 60% worldwide (Hao et al., 2020). Globally, the travel and tourism industry is the most significant contributor to the service sector, and it plays an indispensable role in the world economy (Arbulú et al., 2021). The tourism sector is a vital socio-economic factor for the community (Aman et al., 2019). According to 2018 estimation, tourism and travel sectors helped produce almost 10.42% of the global GDP, and this industry generated a similar share of global employment (Lei et al., 2021). Tourism activities have shown massive resilience and support over the last decade. The COVID-19 pandemic has massively struck travel and tourism and caused adverse effects. Fueling this industry and relative stability for the middle-class people’s growth purpose in Asia and other regions of the world helps expand the tourism industry. Travel and tourism experts expect a significant contribution to world GPD and anticipate a rise of almost 50% in the coming decade (Jucan and Jucan, 2013; Canh and Thanh, 2020).

European countries are the major markets in the global tourism industry and represent one in two trips (50%) worldwide, which accounts for almost 48% of the total outbound tourism (Boluk et al., 2019). The tourism industry is a crucial segment of the service sector. It is the most significant contributing factor and economic driver for the gross domestic product (GDP) for respective countries (Wondirad et al., 2021). According to the data of 2018, the DACH region comprised of Germany (D), Austria (A), and Switzerland (C.H.), and these countries specified their GDO 5.1 trillion dollars (UNWTO, 2019). The service sector in Australia contributed 62.50% to the total Australian GPD. In Germany, the service industry contributed 61.8%, and Switzerland’s service sector contributed 71.4%. The DACH region’s outbound tourism accounted for over 135 million travelers in 2018. Germany is the third-largest spender at 94 billion dollars for tourism activities (Daye et al., 2019; Neuburger and Egger, 2020).

Scholars of tourism studies have paid close attention to perceived socio-economic and the actual contribution of tourism activities to communities of destinations (Lindberg and Johnson, 1997; Mamirkulova et al., 2020; Joo et al., 2021). Inbound tourism creates a profound influence on society, and in conjunction with its positive impacts, tourist arrivals interfere with residents’ economic, cultural and social wellbeing in the tourist destinations (Jordan et al., 2021). However, inbound tourism’s adverse effects have been exacerbated in crises and disasters, such as the emergence of the COVID-19 pandemic (Tambo et al., 2021). At present, the world has faced a global health crisis and economic disaster in the form of the novel virus COVID-19, which struck more than 200 countries and territories (Acter et al., 2020; Agarwal et al., 2021; Lange, 2021). As the ongoing COVID-19 pandemic swept the entire world, many countries and regions imposed border shutdowns and travel restrictions to curb the quick spread of the infectious virus (Jimenez et al., 2020). Richter (2016) debated that appurtenance or re-emergence of infectious viruses is one of the consequences of declining world travel and tourism mobility trends. Globalization and urbanization are driving a quick spread of emerging viruses. However, tourism and travelers’ play a vital role in exacerbating public health crises resulted in the emergence of epidemics and pandemics worldwide (Hall et al., 2020; Hilsenrath, 2020).

Thus, it is essential to quantify and identify the perceived socio-economic and social cost risks of travel and tourism activities in the emergence of the coronavirus outbreak to minimize the adverse impacts on tourism destination regions and cities (Abbas, 2020a; NeJhaddadgar et al., 2020; Su et al., 2020, 2021c). Scholars have paid attention to examine the negative consequences of the COVID-19 on the travel and tourism industry and how to revive this sector to normal condition. Some empirical and qualitative studies have examined the impacts of the global crisis, including natural disasters, on the travel and tourism sectors (Blake and Sinclair, 2003; Kuo et al., 2008; Wang, 2009; Cró and Martins, 2017; Aliperti et al., 2019; Song et al., 2019; SioChong and YukChow, 2020). However, existing literature specified that academia, researchers, and policymakers had paid less attention to how the tourism and travel industry can amplify global crisis events. Such disasters pose adverse impacts on stakeholders’ and residents’ public interest and wellbeing at tourist destinations (Ritchie, 2008). Social media platform plays a positive role for effective communication, and people seek health-related information by using social media from peer groups (Abbas et al., 2019, 2021; Abbas, 2020b,^2021^; NeJhaddadgar et al., 2020; Maqsood et al., 2021; Su et al., 2021c; Zhou et al., 2022). Scott and Laws (2006) described that crisis impacts should be viewed as the interlinked business systems and different stakeholders, which make an attractive tourist destination. Additionally, the key stakeholders and destination residents are crucial in responding to crisis management events at tourist destinations belonging to both private and public sectors (Ritchie, 2008; Karl et al., 2020; Le and Phi, 2021; Sarkar et al., 2021).

The interlinked socio-economic, cultural, political, and psychological consequences of COVID-19 pandemic of this magnitude, the unforeseen trajectories as alternative historical trends might occur, and the old explanatory models with predictive powers may not work in this crisis. Besides, there is enough indication to claim that both academic research and the travel and tourism industry have matured enough to provide sufficient knowledge about how to investigate and efficiently: (i) design and implement global crisis recovery and responsive strategies (McKercher and Chon, 2004). Moreover, (ii) it urges in building resilience to address and manage future crisis events (Hall et al., 2018). The question arises what still lacks is the knowledge about how global crisis can foster economic activities and business industry change. How enterprises can convert the global crisis disruption into innovation transformation and implement research can enable informing, shaping and rethinking, and making efforts to reset the next normal of the travel and tourism industry.

## Social Media Technology Support in Global Health Crisis

With the massive struck of the COVID-19 pandemic, people started using social media more often than usual because they relied on news sources from online sources to receive health information for themselves as well as their family members and loved ones (de Calheiros Velozo and Stauder, 2018; Li et al., 2018). Social media platforms’ usage has become a welcome relief in the health disaster and global crisis during the ongoing COVID-19 pandemic (Zhong et al., 2021). This article holds that analyzing social media usage in the context of global health catastrophes like the COVID-19 pandemic should help disclose the global mental health toll (Lebni et al., 2020). The US Census Bureau surveyed more than 42% of people and identified symptoms of depression and higher anxiety levels in December 2020, which was 11% higher than the previous year. The survey findings of Hazarika reported similar results of COVID-19 mental stress worldwide. When the global health crisis of COVI19 struck, a telephone service supported by Assam police studied 239 callers in April 2020 and found that 46% had anxiety, 22% indicated depression symptoms, and 5% had suicidal thoughts. It was enough evidence to convince the Government to launch a countrywide remote mental health telephonic service to tackle mental health wellbeing. Physical activities could be medicine for non-communicable diseases (Saqib et al., 2020). After easing lockdowns and restrictions on social distancing in December 2020, the telephonic service collected 43,000 people data and found that 9% of people had anxiety symptoms, 4% had depression, and more than 12% of individuals reported stress related to the health crisis posed by the COVID-19 pandemic (Abbott, 2021).

### Social Media, Health-Related News, and Peer Support

Social media has played a positive and indispensable role in providing health information from peer support to the public world (Fang et al., 2020). The concept of peer support refers to the informational support provided by others who are helping to share the experiences to provide health information about the health crisis caused by the COVID-19 pandemic (Tan et al., 2021). The most effective and helpful peer support is assistance matching health-related information needs for social media users (Hosseini et al., 2019). Patients with risk factors need emergency treatment (Firouraghi et al., 2020). The users are eager to seeking information and stay connected with other people, and they share a sense of belonging to the matching group of people (Brewer, 2016). People receive peer support on social media and other online resources that increase self-efficacy and self-esteem and minimizes the risk of self-uncertainty among people (McKenna and Bargh, 1998). Seeking health-related information through social media and social interaction is vital in individuals’ lives who need medical treatment due to health problems (Naslund et al., 2016; Abbas et al., 2021; Sarfraz et al., 2021; Toqeer et al., 2021). Overall, peer support through social media and online resources complements communication desires to retain social connection and reduce the social isolation necessary to manage mental health disorders, depression, anxiety, and secondary trauma (Lin and Kishore, 2021; Yang et al., 2021).

### Social Media Use and Health Behavior

Social media has provided a platform of updated information for the people who seek health-related information about the COVID-19 pandemic (Khazaie et al., 2021; Yoosefi Lebni et al., 2020, 2021). People have faced significant pressure and health threat caused by the coronavirus pandemic, which has increased social media use, as people want to seek accurate health-related information and stay connected with peers, friends, and family (Zhao et al., 2021). Through social media applications, public communication and interaction go beyond personal massages delivery to seeking correct information and the full scope of the COVID-19 pandemic to develop a real sense of virus prevention (Tang et al., 2021). The emergence of the COVID-19 outbreak has changed life patterns in response to preventive measures (Gever et al., 2021). The ongoing global health crisis has developed a strong sense of coronavirus contagious disease prevention. It might promote health behavior changes, such as maintaining social distancing, using sanitizer, wearing masks, and washing hands. The health behavior theories explain the health behavior model (Rosenstock, 2005; Pouresmaeil et al., 2019; Fattahi et al., 2020), which describes why individuals fail to adopt preventive measures or screening tests for the early detection of infectious disease (Carpenter, 2010). The health behavior model helps understand the useful strategies to improve people’s health behavior, like adherence to medical treatment against the disease (Jones et al., 2014). The HBM explains that people start to be involved in health-related behavior when people perceive susceptibility to a contagious disease, which has severe health consequences. Its benefits to health-related human behavior outweigh the barriers (Castonguay et al., 2016).

## Global Healthcare Systems and Crisis Management

The appearance and recurrence of infectious diseases result in a decline in economic and tourism activities worldwide (Piccinelli et al., 2021). The advent of the COVID-19 outbreak posed pressure on healthcare systems and quickly instigated disruptions to travel and tourism destinations, healthcare systems, and the world economy (Bausch et al., 2020). The ongoing global crisis resulted in numerous problems and crisis management challenges unfolding yet (Farzanegan et al., 2020). This study investigates strategic retorts on self-protective measures on travel and tourism activities to contain the virus’s rapid spread and minimize the adverse impacts on mental health and economic losses (Crespí-Cladera et al., 2021). This article primarily focuses on crisis management and disruption in the travel and tourism sector and measures to revive business activities to the next normal (Zenker and Kock, 2020). The study debates that intervention strategy controls the COVID-19 quick spread with hands-on management of the tourism industry crisis. The support of the respective governments can help revive the economic activities and service sectors, including travel and tourism, by implementing collaborations and the scientific contribution to reset the business industry to a normal situation (Pereira et al., 2020). Managing a crisis and challenging disaster is a thought-provoking and complex theme to address the emerged problems. Crisis management needs to be effectively coordinated through an interdisciplinary and multidisciplinary approach to large-scale institutional, organizational and individual responses to address this critical issue.

Each crisis event offers positive aspects and patrimonial opportunities in perceiving and responding to a global challenge or crisis events. Resilience effectively helps to deal with challenging circumstances and crisis management (Wut et al., 2021). Crisis management in threatening and stimulating conditions provide numerous opportunities in capitalizing on new economic horizons. Thus, enterprises’, business experts, policymakers, and decision-makers need to implement reasonable practical and innovative skills and creative, intelligent business plans to handle the crisis positively. Hence, it helps in reviving the travel and tourism industry. It helps explore and proficiently: (1) design and implement crisis management and practical strategies. Still, (2) establishing resilience to resolve and manage the events of future crises (Wong et al., 2021; Wut et al., 2021; Zheng et al., 2021). The current study aims to address these identified gaps and critically review the existing and emerging literature to help university circles, investigators’ professionals, and scholars alike to better understand, accomplish and valorize the travel and tourism industry impact and the COVID-19 pandemic affordance setting. Thus, to attain this end, this study discusses how the COVID-19 global challenges urge to design and implement crisis management strategies that might be a transformational opportunity to raise questions about transformational opportunities and recovery strategies associated with the virus circumstances.

## Challenges to Travel and Tourism Industry in the Wake of a Global Health Crisis

The emergence of the COVID-19 outbreak has struck across the globe, affecting almost every region worldwide. In response to a worldwide health crisis, many countries and regions imposed border shutdown and travel restrictions to combat the infectious virus’s quick transmission. The ongoing pandemic COVID-19 has caused a significant decline to all segments of the world economy; however, service sector faced a massive hit, including the travel and tourism industry, which is the most critical contributor to the service industry worldwide. The appearance of the COVID-19 virus resulted in a global health crisis and economic crunch and posed a significant decline in the travel and tourism industry. As of March 9, 2021, there were more than 117.989 million confirmed patients of the COVID-19 disease, with more than 2.617 million death toll attributed to the ongoing coronavirus pandemic, making it the deadliest pandemic in human history. There were more than 93.641 successful recovered patients as of March 9, 2021, worldwide (Wang et al., 2020).

### Europe

Europe was the epicenter, and there was a massive struck of the COVID-19 pandemic in many European countries. There was a falling trend in daily new cases in most countries earlier, but many countries are now reporting a rise in the ongoing COVID-19 pandemic. Poland, France, Italy, Germany, and the Czech Republic have recorded the highest number of infected patients in recent weeks. The worst-affected countries imposed border shutdowns and restrictions on social gatherings at public places and tightened preventive measures to combat the disease’s quick spread. The rise in the number of infected cases was due to relaxation on restrictions. As of March 13, 2020, the number of new confirmed cases crossed China’s total confirmed patients. The World Health Organization instigated considering the European region as the active epicenter of the COVID-19 pandemic. In the first wave, cases by country in the region of Europe had doubled within 3–4 days, with some countries, mostly those states at earlier phases of the virus detection, reported doubling of cases every 2 days. As of March 17, 2020, each country in Europe had reported a confirmed patient of the COVID-19.

As of March 18, 2020, the European countries imposed lockdowns and restrictions on social gatherings, and more than 250 million European people faced lockdowns against COVID-19 protective measures to contain the quick spread. As of March 01, 2021, there were more than 37.75 million confirmed COVID-19 cases across the entire European region since the first confirmed infected patients were reported in France on January 25, 2020 (Aqeel et al., 2021; Shuja et al., 2020). Europe reported almost two million new cases in the week ending November 8, the highest record of confirmed cases in a single week. Since the end of August 2020, Europe reported a distinct rise in the number of newly infected patients. This ongoing global health crisis posed a sharp decline in travel and tourism activities worldwide. The daily new cases of the COVID-19 (based on the 7-Days moving average) as of March 8, 2021 indicated that North America and the U.S. are the most affected countries, and the ongoing pandemic has massively struck in these countries. South America, France, Germany, India, and the United Kingdom are also positively affected by infectious disease. In China, the situation remains under control, as they have successfully contained the spread of the virus in the country. These infections of the pandemic COVID-19 virus has resulted in psychological issues, fear and anxiety among employees of the hospitality and tourism industry.

### Global Effects of the COVID-19 Pandemic

As of March 9, 2021, the WHO has confirmed global cases of over 117.99 million infected by the COVID-19 virus. More than 2.62 million deaths were attributed to the COVID-19 pandemic, making it one of the lethal pandemics in human history. The global preventive measures to the COVID-19 pandemic have resulted in significant disruptions in social, economic, and health systems. It has caused the most significant recession worldwide since the great depression, which began as the most severe economic depression during the 1930s. The ongoing pandemic has led to the cancelation or postponement of economic events, widespread supply chain shortage exacerbated by consumers’ panic buying, agricultural disruptions and food items shortage, and low emissions of pollutants and greenhouse gasses. Governments imposed restrictions on face-to-face education and closed partially or fully the public areas and educational institutions to combat the virus damages. The COVID-19 outbreak resulted in an infodemic through mass media and social media platforms, eroded public trust, impeded the virus’s restraint, and outlived the pandemic itself. The emergence of the COVID-19 has raised numerous problems, such as health equity, the balance between individuals’ rights and public health imperatives, and racial and geographic discrimination.

**FIGURE 1:**
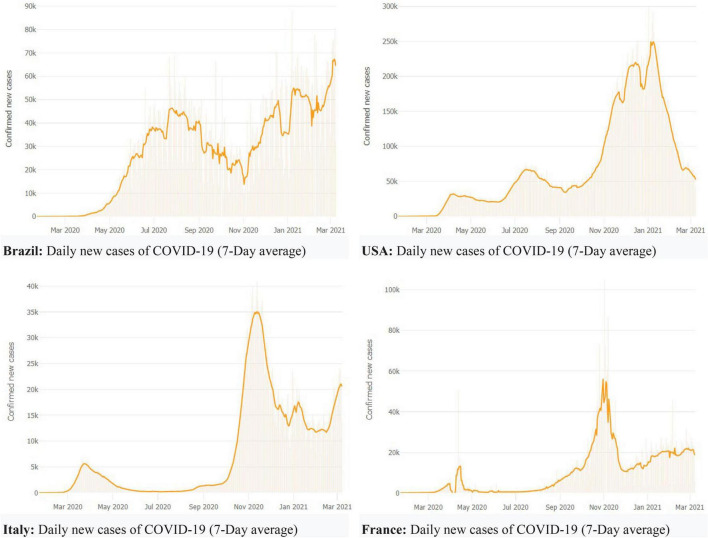
The trend of COVID-19 confirmed cases in Italy and France, as of March 1, 2021.

Table 1 indicates the cases and mortality ratio of the COVID-19 by most patients in the top affected countries, as of March 9, 2021, worldwide. The WHO statistics reported that the United States had the most number of confirmed cases, 29,038,631 death toll 525,752, and a case-fatality ratio of 1.80% in the world. India is the second most victim of the COVID-19 confirmed patients and reported 11,244,786, with a death toll of 157,930 and a case-fatality rate of 1.40%. Brazil is the third most affected country and declared confirmed 11,051,665 cases, death toll 266,398, and case fatality ratio 2.40%. Russia reported total cases of 4,284,408, deaths 87,985, with a case fatality ratio of 2.10%, and the United Kingdom reported 4,235,989 confirmed patients of the coronavirus, death toll 124,801, with a case fatality rate of 2.90%, correspondingly. See Figure 1 for details about daily new cases of the COVID-19 of the selected countries and regions based on confirmed cases and the most affected country’s mortality rate worldwide. See Table 1 for further details. The increasing cases and infection of the coronavirus has resulted in psychological issues, fear and anxiety among employees of the hospitality and tourism industry, which resulted in negative impacts on employees performance.

**TABLE 1:** COVID-19: confirmed cases and mortality by the most affected countries, as of March 9, 2021.

Country	Confirmed	Deaths	Case-fatality %	Deaths/100k pop.
United States	29,038,631	525,752	1.80	160.7
India	11,244,786	157,930	1.40	11.68
Brazil	11,051,665	266,398	2.40	127.18
Russia	4,284,408	87,985	2.10	60.9
United Kingdom	4,235,989	124,801	2.90	187.7
France	3,969,612	89,090	2.20	133
Spain	3,160,970	71,436	2.30	152.89
Italy	3,081,368	100,103	3.20	165.65
Turkey	2,793,632	29,094	1.00	35.34
Germany	2,513,784	72,236	2.90	87.11
Colombia	2,278,861	60,598	2.70	122.05
Argentina	2,154,694	53,121	2.50	119.39
Mexico	2,130,477	190,923	9.00	151.3
Poland	1,801,083	45,317	2.50	119.32
Iran	1,698,005	60,786	3.60	74.31
South Africa	1,521,706	50,803	3.30	87.93
Ukraine	1,455,421	28,616	2.00	64.13
Indonesia	1,386,556	37,547	2.70	14.03
Peru	1,371,176	47,854	3.50	149.59
Czechia	1,325,291	21,882	1.70	205.93
Netherlands	1,139,102	15,990	1.40	92.8
Canada	896,247	22,271	2.50	60.1
Chile	860,533	21,163	2.50	112.99

*Source: John Hopkins University CSSE COVID-19 Data.*

The COVID-19 data indicated that the number of deaths due to daily new cases of the COVID-19 of the selected countries and regions cuased challenges. There was a quick rise in the number of patients from March to April 2020, and new patients significantly decreased from May to August 2020. From September 2020 to February 2021, there was a rising trend of new cases worldwide. The number of new cases has sharply decreased, but the pandemic is still growing across the world. Some countries have faced massive spread, while some nations have controlled the infection effectively. The increasing cases and infection of the coronavirus has resulted in psychological issues, fear and anxiety among employees of the hospitality and tourism industry, which resulted in negative impacts on employees performance. Globally, the COVID-19 mortality rate is almost 3.4%, which shows a ratio of the reported confirmed cases has died. By comparison, the seasonal flu usually causes death far fewer than 1% of the infected patients globally—initially, the WHO has mentioned a 2% mortality rate. However, the WHO specified that the estimation of 2% was much early, and provisional forecasting has been changed over time. Surveillance indicated an increasing trend within China, but it also reported a growing number of infected patients worldwide (D’Amico et al., 2020).

Table 2 specifies a case and mortality ratio by most affected countries with patients of the COVID-19, as of March 9, 2021. The World Health Organization declared that the United States was the most affected country with the highest death toll. The U.S. reported confirmed cases, 29,038,631 deaths, 525,752, and a case-fatality ratio of 1.80%. Brazil is the second most affected country with a death toll and reported a total of 11,051,665 cases, a death toll of 266,398, and a case fatality ratio of 2.40%. Mexico is the third-highest death toll country and reported 2,130,477 cases, with total deaths 190,923 and a case fatality rate of 9.00%. In comparison, India is the fourth nation with the highest recorded deaths due to the COVID-19 attack.

**TABLE 2:** COVID-19: cases and mortality (Deaths) by the most affected countries, as of March 9, 2021.

Country	Confirmed	Deaths	Case-fatality %	Deaths/100k pop.
United States	29,038,631	525,752	1.80	160.7
Brazil	11,051,665	266,398	2.40	127.18
Mexico	2,130,477	190,923	9.00	151.3
India	11,244,786	157,930	1.40	11.68
United Kingdom	4,235,989	124,801	2.90	187.7
Italy	3,081,368	100,103	3.20	165.65
France	3,969,612	89,090	2.20	133
Russia	4,284,408	87,985	2.10	60.9
Germany	2,513,784	72,236	2.90	87.11
Spain	3,160,970	71,436	2.30	152.89
Iran	1,698,005	60,786	3.60	74.31
Colombia	2,278,861	60,598	2.70	122.05
Argentina	2,154,694	53,121	2.50	119.39
South Africa	1,521,706	50,803	3.30	87.93
Peru	1,371,176	47,854	3.50	149.59
Poland	1,801,083	45,317	2.50	119.32
Indonesia	1,386,556	37,547	2.70	14.03
Turkey	2,793,632	29,094	1.00	35.34
Ukraine	1,455,421	28,616	2.00	64.13
Belgium	789,008	22,292	2.80	195.17
Canada	896,247	22,271	2.50	60.1
Czechia	1,325,291	21,882	1.70	205.93
Chile	860,533	21,163	2.50	112.99

*Source: John Hopkins University CSSE COVID-19 Data.*

There were 11,244,786 cases, with a death toll of 157,930 and a case-fatality rate of 1.40%. The United Kingdom reported 4,235,989 established patients of the coronavirus, death toll 124,801, with a case fatality rate of 2.90%. Russia reported total cases of 4,284,408, deaths 87,985, with a case fatality ratio of 2.10%. France had shown actual confirmed patients of 3,969,612, with a death toll of 89,090 and a case fatality rate of 2.20%. Germany reported 2,513,784 established patients of COVID-19 disease, with total deaths 72,236 and a case fatality ratio of 2.90%, correspondingly. Figure 1 shows the trend of the COVID-19 confirmed cases in some countries, including Italy and France, from March 1, 2020.

Figure 1 specifies that Brazil has indicated a rising trend in daily new cases on a 7-day average. The number of daily cases has decreased in the U.S. after January 2021. France has shown an increasing trend in recording daily new patients, and Italy has faced a rise in new cases in February 2021. Many countries are facing a second and third wave of the ongoing pandemic COVID-19 worldwide. Europe has become the epicenter, and there were around 38 million cases of the virus. The global struck of the COVID-19 has caused substantial disruption to the service industry, including the travel and tourism sector, which is a significant contributor to the service industry worldwide.

The global health crisis of the COVID-19 has massively disrupted global economies and posed substantial damage to the travel and tourism industry worldwide. The United Nations World Tourism Organization estimates that international visitors declined by 60–80 percent by the end of 2020. The consequences results showed potential economic damages of $0.9–1.20 trillion for the global tourism industry. Many resorts and cities reported an 80–90 percent drop in planned travel and tourism. Travel restrictions on tourists are conflicting and unilateral, and many global tourist attractions, such as amusement parks, sports venues, and museums, are under restrictions. UNWTO stipulates that the number of international visitors will fall by 65% in the first half of 2020. Air passenger travel reports are down more than 60% globally.

Table 3 specifies that Yemen has indicated the highest case-fatality ratio (CFR = 25%), as of March 12, 2021. They have reported 2,667 infected cases, a death toll of 667, and deaths per 100K population is 2.34. Mexico has reported the second-highest case-fatality ratio (CFR = 9%). There are 2,150,955 confirmed infected patients with a death toll of 193,152, and per 100K population, deaths are 153.06. Syria reported case-fatality ratio (CFR = 6.7%), and Sudan reported case-fatality ratio (CFR = 6.3%).

**TABLE 3:** COVID-19: cases and mortality (Case-Fatality Ratio) by the most affected countries.

Country	Confirmed	Deaths	Case-fatality %	Deaths/100k pop.
Yemen	2,667	667	25.0	2.34
Mexico	2,150,955	193,152	9.0	153.06
Syria	16,257	1,085	6.7	6.42
Sudan	30,686	1,940	6.3	4.64
Egypt	189,000	11,169	5.9	11.35
Ecuador	297,957	16,128	5.4	94.40
China	101,225	4,839	4.8	0.35
Bolivia	257,240	11,903	4.6	104.84
Afghanistan	55,917	2,451	4.4	6.59
Liberia	2,026	85	4.2	1.76
Bulgaria	272,700	11,094	4.1	157.94
Zimbabwe	36,377	1,492	4.1	10.33
Mali	8,782	359	4.1	1.88
Tanzania	509	21	4.1	0.04
Comoros	3,615	146	4.0	17.54
Bosnia and H.*	140,990	5,410	3.8	162.76
Eswatini	17,215	659	3.8	58.00
Somalia	8,820	338	3.8	2.25
Niger	4,853	180	3.7	0.80
Guatemala	181,143	6,531	3.6	37.87
Chad	4,246	151	3.6	0.98
Peru	1,387,457	48,323	3.5	151.06
Iran	1,723,470	61,016	3.5	74.59

John Hopkins University CSSE COVID-19 Data. *Bosnia and Herzegovina.

Table 3 specifies COVID-19 cases and mortality (Case-Fatality Ratio) declared by the most affected countries, as of March 12, 2021. Table 3 sets that Yemen has indicated the highest case-fatality ratio (CFR = 25%), as of March 12, 2021. They have reported 2,667 infected cases, a death toll of 667, and deaths per 100K population is 2.34.

Table 4 indicates the infected patients with COVID-19 positive scenario worldwide. Showing the transmission, new cases, total deaths, overall recoveries worldwide. Table 4 designates a detailed COVD-19’s analysis with its global spread, recoveries, total deaths, and serious/critical COVID-19 patients as of October 09, 2021. The current COVID-19 virus has caused domestic, regional and global health issues worldwide (Aman et al., 2021; Aqeel et al., 2021; Azadi et al., 2021; Local Burden of Disease HIVC, 2021; Paulson et al., 2021; Soroush et al., 2021; Wang et al., 2021).

**TABLE 4:** COVID-19 infected cases in top 20 countries worldwide, as of October 09, 2021.

	Country,	Total	New	Total	New	Total	New	Serious,	Total	Tests/	Population

	other	cases	cases	deaths	deaths	recovered	recovered	critical	tests	1M pop	
	World	237,979,622	450,499	4,856,844	7,666	215,119,880	448,807	83,334			
1	United States	45,136,852	107,097	732,477	1,937	34,577,516	98,385	17,497	656,553,737	1,968,860	333,468,970
2	India	33,934,335	19,870	450,408	248	33,240,703	23,066	8,944	580,043,190	415,136	1,397,235,287
3	Brazil	21,550,730	18,172	600,493	628	20,665,273	58,917	8,318	63,776,166	297,354	214,479,029
4	United Kingdom	8,085,109	41,024	137,564	147	6,593,525	32,842	808	311,519,071	4,558,427	68,339,166
5	Russia	7,717,356	27,246	214,485	936	6,819,796	20,566	2,300	195,600,000	1,339,597	146,014,021
6	Turkey	7,387,537	30,201	65,778	188	6,843,487	28,167	633	89,154,839	1,042,851	85,491,480
7	France	7,047,786	4,470	117,029	38	6,832,528	7,380	1,200	146,046,715	2,231,182	65,457,114
8	Iran	5,683,980	9,897	122,197	185	5,186,096	16,097	5,451	32,619,228	382,147	85,357,709
9	Argentina	5,265,058	753	115,444	28	5,130,084	1,450	981	24,252,818	530,435	45,722,524
10	Spain	4,973,619	2,309	86,778	77	4,800,693	3,190	550	65,473,038	1,399,660	46,777,803
11	Colombia	4,969,131	1,607	126,552	35	4,812,120	1,808	342	25,895,959	502,136	51,571,586
12	Italy	4,695,290	3,022	131,228	30	4,478,137	4,234	383	94,716,158	1,569,480	60,348,769
13	Germany	4,316,499	9,742	94,988	78	4,074,300	9,700	1,336	73,348,901	871,909	84,124,457
14	Indonesia	4,225,871	1,384	142,560	66	4,057,760	3,514		41,116,704	148,331	277,196,503
15	Mexico	3,707,234	7,613	281,121	514	3,063,722	6,012	4,798	10,838,697	82,958	130,652,960
16	Poland	2,918,862	1,894	75,835	32	2,666,589	760	203	21,204,795	561,064	37,793,902
17	South Africa	2,910,681	924	88,236	132	2,789,432	1,302	546	17,931,034	297,571	60,257,979
18	Philippines	2,643,440	10,616	39,232	191	2,485,858	7,490	3,194	21,657,259	194,353	111,432,443
19	Ukraine	2,514,005	16,362	58,081	241	2,282,482	4,720	177	13,164,600	303,335	43,399,511
20	Malaysia	2,323,478	9,751	27,191	78	2,170,289	12,724	792	31,182,322	948,009	32,892,425

*Source: WHO and JHU CSSE COVID-19 Data. The yellow text shows new cases and the red text new deaths. The text in yellow color shows new COVID-199 cases, red shows new deaths due to the COVID-19, and the green color text shows new recovered COVID-19 patients in the countries reporting the highest number of cases.*

Table 5 shows the COVID-19 confirmed total cases and death toll, cases per million, deaths per million, and total population by different regions, in absolute facts and figures and ratio per million inhabitants, as of February 23, 2021. Table 4 shows that European Union states and the United Kingdom reported total infected cases 25,800,811, with a death toll of 646,721, cases per million 50,062, and death number per million 1,255. North America declared total cases 29,617,270, deaths 532,951, cases per million 80,306 and deaths per million 1,445. Similarly, South America recorded COVID-19 cases 17,451,165, deaths 455,006, cases per million 40,596, and deaths per million 1,058, respectively.

**TABLE 5:** COVID-19 confirmed cases and death toll by different regions as of February 23, 2021.

Region/Country	Total cases	Deaths	Cases/mil.	Deaths/mil.	Population
European Union and United Kingdom	25,800,811	646,721	50,062	1,255	515,379,780
North America	29,617,270	532,951	80,306	1,445	368,807,085
South America	17,451,165	455,006	40,596	1,058	429,874,454
South Asia	12,550,114	182,791	6,761	98	1,856,376,616
Central America	2,996,982	201,641	16,680	1,122	179,670,186
Middle East	5,283,101	101,945	20,242	391	260,991,715
Russia and Central Asia	5,325,852	97,248	22,492	411	236,793,935
Other Europe	5,134,459	87,252	31,009	527	165,581,143
Sub-Saharan Africa	2,699,235	69,613	2,375	61	1,136,650,011
Oceania, islands in East Asia	2,314,737	55,158	4,010	96	577,280,423
North Africa	1,136,396	31,858	5,632	158	201,785,909
East Asia	708,605	10,811	399	6	1,777,630,472
Caribbean	546,776	6,719	12,629	155	43,295,072
**Total (or Average)**	**111,565,503**	**2,479,714**	**14,395**	**320**	**7,750,116,801**

*Source: John Hopkins University CSSE COVID-19 Data.*

## Risk Perception and Crisis Management Amid COVID-19 Pandemic

The opinion of travel and tourism research scholars about tourism and crisis falls into two contexts: perception of risks at individuals’ level on tourism demand and crisis management in the pandemic at the collective levels, such as supply side. Research on the perceived risk perception associated with the travel and tourism industry focuses on tourists’ perspectives rather than considering destination communities’ views. The notion of perceived risks in tourism is linked primarily with consumer behavior research (Fu et al., 2021; Im et al., 2021; Ma et al., 2021). Scholars have examined safety and risk perception issues typically from tourists’ perspectives, seeking to determine why tourists perceive risk factors differently and what types of elements impact travelers’ risk perception (Lepp and Gibson, 2003; Gössling et al., 2012; Wang et al., 2019). Tourism-related risk perception may indicate an association with abnormal circumstances, such as war, terrorism, social instability, political, criminal, or health-related public concerns. Perceived risks or anxiety can influence tourists to avoid specific regions or cities. Still, travelers might drop visiting such places due to past unpleasant experiences, novelty-seeking behaviors (Sharifpour et al., 2014; Wong et al., 2014; García-Villaverde et al., 2021; Halder and Sarda, 2021), or their cultural orientations (Reisinger and Mavondo, 2006; Cuomo et al., 2021). From the perspective of the tourism supply side, the previous studies on the travel and tourism industry have taken crises and pandemics as a dominant theme, as it affects tourists’ psychology and mental health at a large scale worldwide. The existing research studies have focused on examining the effects on tourism demand during various global crises, such as the September 11 attacks on the U.S. in 2001, earthquakes, other terrorist activities (Seabra et al., 2020), the severe acute respiratory syndrome (SARS) outbreak in 2003 (Wang, 2009), and the swine flu (H1N1) pandemic in 2009 and the global economic crisis from 2007 to 2008 (Page et al., 2011), tourists boycotts and the COVID-19 outbreak (Yu et al., 2020).

Another study explored the swine flu impact on destination planning by considering perceived risks through media coverage on influenza outbreaks (Page et al., 2011). Nevertheless, Ritchie and Jiang (2019) reviewed 142 published studies related to travel and tourism crisis management. They identified responses and recovery strategies, preventive measures of crisis and planning practices, a lack of in-depth theoretical and methodological evaluations of crises in the travel and tourism industry (Ritchie and Jiang, 2019). Some researchers emphasize that crisis management in the tourism industry should consider destination residents’ welfare. With the expansion of industry-related infrastructure, the speedy growth of the tourism sector has posed environmental crises in some destination cities and regions (Lukashina et al., 1996; Ma et al., 2020; Lee and Chen, 2021). The literature documents that tourist destination residents are aware of tourism activities’ positive economic contribution and other associated adverse socio-environmental risk factors affecting local communities’ livelihood (Schmidt et al., 2014; Bires and Raj, 2020; Pham, 2020). However, risk perception and reactions differ among various clusters of destination residents (Antunes et al., 2020). Easterling (2004), Scott and Laws (2006), Abbas (2020b), Mubeen et al. (2021a), Aman et al. (2022), and Mamirkulova et al. (2022) paid precise attention to identifying destination residents’ interests and their representatives to manage a tourist place, including tourism crisis management (Easterling, 2004; Cartier and Taylor, 2020; McCabe and Qiao, 2020). Eitzinger and Wiedemann (2007) emphasized investigating residents’ risk perception because their understandings are based on their specific experiences that differ from tourist destinations. With the appearance of the COVID-19 global crisis, more than 200 territories and countries governments have shut down borders and imposed unprecedented restrictions on social gatherings, movements (Yang et al., 2020). This pandemic has changed their populations’ behavior, and economic activities have decreased enormously worldwide. Besides, tourists’ health risks have increased the concern that infected tourists can spread the lethal infectious disease to destination residents and communities (Gautret et al., 2012). In the emergence of a global pandemic, international travel spreads infectious disease quickly and brings health-related threats to populated urban areas worldwide (Ritchie and Jiang, 2019; Zheng et al., 2021). Travel within communities increases higher risks of transmission of the COVID-19 fatal illness and other respiratory viruses, such as seasonal influenza (MacIntyre, 2020).

## COVID-19 Settings, Travel, and Tourism Industry: Challenges and Recovery Strategies

Scholars have focused on investigating, gauging, and predicting the consequences of coronavirus on the tourism sector is vital to eliminate health risks, formulate, monitor, and improve responsive strategies. However, research concentrating on the characteristics and effects of crises rather than their structural roots often obscures and stabilizes the conditions and inevitable social structures under which crises arise (Barrios, 2017). Examining the actual root causes of the COVID-19 impact can last longer to cross the tourism and leisure research boundaries and scope. However, the latter needs further investigation and challenge the “environment” and structure of tourism, enabling and sometimes accelerating the global spread of COVID-19. Deplorably, economists restrain the idea that the pandemic stems purely from natural events outside the economic system (Nowlin, 2017). However, considering COVID-19 as an external shock and phenomenon unrelated to the structure of socio-economic factors and values can continue and strengthen the virus roots of the post-COVID-19 era and limit the process of change and transformation. The coronavirus pandemic is the global crisis that is associated with economized societies having roots in growth patterns. Tourism requires traveling, travel and leisure growth patterns, and evaluation are the major contributors to current socio-economic systems that accelerate the quick spread and further consequences of the lethal virus. Tourism is responsible for a highly interconnected global world, waste and climate change, pollution, national, international, regional economic growth, capitalism superiority values and economic activities decision-making, and policymaking and politics formulation. The increasing growth in climate change is responsible for more outbreaks; epidemics and pandemics are more commonly expected in the future, highlighting a vicious circle of forces and interwoven nature between physical, biological, and socio-economic systems (Sands, 2019).

Besides, economic systems and mindsets have contributed to the ongoing pandemic and kept guiding and shaping responses and recovery strategies tailored by people, business houses, institutions, and governments. Economic priorities to maintain business stability, jobs, revival, and recovery to the old average economic growth are the main agenda and driving business professionals, policymakers, practitioners, and governments. For instance, financial support with tax relief and subsidies to tourism companies and employees. Scholars have debated on relaxing restrictions to reopen and re-start economic activities at the cost of the second and third wave of the pandemic COVID-19 to increase human lives’ risks. Similarly, people have started to panic buying and consumption through online buying experiences, such as traveling, drinking, dining, and virtual entertainment. The lockdowns demonstrate public preference, persistence, and fear of losing traditional lifestyle and consumerism deemed fit for their happiness and success. The research on COVID-19 tourism reinforces similar mindsets. Scholars have investigated the economic effects of COVID-19 on the tourism industry and traded them off to socio-cultural, biological, and socio-economic impacts. Research focusing on measuring and predicting when tourism will revive and tourists start traveling like a normal situation before and when we can achieve the old traditional targets. Globally, governments are trying to manage the crisis to avoid economic losses on a large scale. They are determined to become first in reopening borders and business activities, including travel and tourism. Financial markets, cash liquidity, and economic survival equally press multinational companies and small tourism firms. They also look forward to receiving help to resume their business operations on the old patterns and business models. Discussion and research rely on trading between financial advantages and economic losses in exchange for morals, humanoid lives, human rights, and ethics.

In general, research, our education, socio-economic and political systems, which shape and each other, frame our mindset on how to conduct research, measurement, understanding, response, and recovery from the COVID-19 global crisis. Subsequently, we have transformed the COVID-19 outbreak from the spread of biological viruses to the financial crisis contagion. We have recently initiated an economic revival race to rebuild the old model based on financial competitiveness. Tourism and leisure research should undertake more responsibility to inform, drive and lead sustainable features to avoid such kinds of perpetuations. To this end, COVID-19 tourism and leisure industry research should not be seen, executed, directed, and used solely as a helpful tool to help restore the traditional old business models. On the contrary, COVID-19 tourism and leisure research should also set challenges to business growth models and assumptions, which have led to existing circumstances and enabled our business phenomena to reimagine and reset the travel and tourism industry to gain the next normal. It is helpful in capitalizing opportunities in the crisis for escaping the unsuitable truism-paradigms paths (Gössling et al., 2020; Hall et al., 2020; Higgins-Desbiolles, 2020; Ioannides and Gyimóthy, 2020). The covid-19 travel, tourism, and leisure research need to assume responsibility on criticism to epistemic and ontological basics, practicalities, and assumptions, which helps underpin the prevailing scientific and technological growth paradigms (Brodbeck, 2019). It should also analyze and challenge mechanisms, patterns, and systems, which help sustain the evolution of deleterious and unsustainable tourism (Higgins-Desbiolles, 2020). However, in order to restore and transform the travel and tourism industry, its social and economic system, tourism research should assume responsibility to find ways, support, and perspectives of research, knowledge, awareness, and development. COVID-19 tourism research should also stimulate, motivate and inform all tourism stakeholders to adopt new ways of existing, doing things, and politicalizing to regain the old successful business growth models.

## Indispensable Role of Technology in Combating COVID-19 Pandemic

Technological applications and Social media platforms have played an indispensable role in fostering economic growth. In the advent of the global health and financial crises, technology remains at the front to provide the core of solutions to combat the adverse of the COVID-19 on international economic activities, including the leisure and tourism industry. Technological innovations have played an essential role in healthcare systems to treat patients. It helps reopen the world economy and tourism sectors, such as social distancing, crowd controlling technologies, prominent big-data use for quick and real-time managerial decision making, identity controls, and digital health passports. It is useful to seeking peer support and health-related information through social media, human robots delivery system materials, robotized-AI touchless mechanism of service delivery, neutering and disinfecting public spaces, measuring and detecting human body temperature, and proving security and safety measures. In the time of the global pandemic, technology offers endless support, such as panacea to human-driven needs for normalizing surveillance under the COVID-19 circumstances to precise and safeguard the safety of human health, to gather information, and analyze personal data for timely decision-making. Though the COVID-19 travel and tourism research cannot prevent technology advancements, it should combat this technological Trojan horse from within to ask questions and resetting its drive, design, formulation, affordance, and ethics interpretation and applications. Technology is made up of a unique affordance, and its development and expression are determined by the institutional logic of design, implementation, and use of technological knowledge (Zuboff, 2015).

The coronavirus Tourism research can merely explore and advance COVID-19 information and technical capabilities to gather, examine, and use big data sets. It helps to understand better, predict, control, and modify human behavior, such as that of tourists and employees, thereby generating revenue, economic growth, and market control (Zuboff, 2015). However, such research-based on the tourism industry will only further support producing the daily data, which imprints the main objectives of the internal components of organizational and institutional life and the commercialization strategy. Technology has always played a role as an enabler, a catalyst for change and innovation, a disruptor of the travel and tourism industry, and a tool for building tourism resilience in the global crisis. In the advent of the COVID-19 pandemic, the crisis strengthens the role of technology in tourism recovery and reimagining while reinforcing the existing paradigms in the evolution of e-tourism. Trends and the espousal of smart tourism destinations and travel services, artificial intelligence, digital advances, and robotics are now accelerating in combating COVID-19 tourism implications. The research of COVID-19 tourism should assume responsibility in reimagining and reshaping the travel purpose, use, and means of these technological advancements, which have primarily shaped how our societies and global economies are transforming and how tourism activities are being implemented, managed, and developed with the help of COVID-19 crisis (Constantiou and Kallinikos, 2015).

## Discussion and Conclusion

The COVID-19 pandemic resulted in long-lasting economic, socio-cultural, and adverse psychological consequences on different stakeholders. Scholars feared that some impacts of the pandemic would stay for several years ahead. This study primarily aims to encourage and motivate the tourism and leisure industry scholars to analyze and assume responsibility to consider the COVID-19 pandemic as the transformational opportunity for crisis management. They need to reform their minds to design and conduct research. Technological innovations are helpful to handle the global crisis, and tourism institutions should reset and redesign their metrics and standards to motivate and evaluate the research purpose, role, and consequences related to tourism and the leisure industry. Besides, crises create opportunities to accelerating technological advancements, changes, and innovation. Technological advances, tools, and social media use play a vital role in driving economic stability and growth. In the digital health and financial crisis contexts, technology applications are forefront to address the undesirable COVID-19 impacts on business activities, such as travel, leisure, and tourism. Technological innovation and advancements are essential in the global health care systems to treat patients. Technology supports to reopen the world economy and tourism industry, for instance, social alienation, crowd control technologies, prominent big data use for rapid and real-time management decision-making, identity control, and digital health passports (Colombo et al., 2016). Although, these preventive measures are helpful to combat the COVID-19 tourism impacts. They are not inevitable and unquestionable to reshape and reallocate humans’ actual needs and purposeful values. Scholars should assume the responsibility to safeguard that COVID-19 tourism research ensures the latter.

The study findings can inspire travel and tourism firms to monitor, understand, and manage the crisis evolution scenarios and execute preventive anti-CIVID-19 pandemic strategies to strive for the tourism industry revival to the next normal and revitalization. The tourism research practitioners and managers can adopt the COVID-19 crisis management framework to manage tourists’ mental health-related catastrophes in broader contexts that enhance the study findings’ generalizability. The travel and tourism industry in China became the first victim of the COVID-19 pandemic ramifications. They adopted timely preventive safety measures to contain and reduce the economic losses and implemented safeguard policies to secure tourists and employees. The tourism industry implemented social responsibilities, participated in anti-pandemic campaigns during social gathering restrictions, and elongated lockdown. After witnessing recoveries and positive signs, the travel and tourism industry adopted a series of health guidelines and innovative measures to revitalize its performance. However, the COVID-19 tourism ramifications remain uncertain as the leisure industry’s economic crisis is existentially intimidating.

Crisis management strengthens the tourism and leisure industry and aims to examine: (1) designing and implementing global crisis and hands-on strategies. Besides, (2) to establish resilience in resolving and managing the crisis events for future pandemics (Wong et al., 2021; Wut et al., 2021; Zheng et al., 2021). This study aimed to address the recognized literature gaps and critically review the existing and emerging literature to help university circles, investigators’ professionals, and scholars alike to better understand, accomplish and valorize the travel and tourism industry impact and the COVID-19 pandemic affordance setting. Thus, to attain this end, this study discusses how the COVID-19 global challenges urge to design and implement crisis management strategies that might be a transformational opportunity to raise questions about transformational opportunities and recovery strategies associated with the circumstances of the coronavirus pandemic. This study aims to recognize fundamental values, enterprises, and pre-assumptions unified with the travel and leisure industry. It helps revive tourism, academia, and researchers to bring breakthroughs in introducing frontier areas of practical research. The present article debated the actual impacts of the next phase, visitor behavior, and experience. The travel and tourism industry’s principal shareholders include tourism demands and enterprises’ that organize tourist places and decision-makers. They have response experiences, rehabilitation, and relocation of tourism events.

This article offers helpful awareness of COVID-19 effects on tourism and the practical vitalizing to tourism research. The study steps forward to discuss significant COVID-19 tourism impacts, public behaviors, understandings, experiences that major tourism stakeholders (tourism demand, supply, and firms or enterprises managing tourist destinations and policymakers) have experienced during the pandemic appearance. The study analysis provides an insightful understanding and overview of the impacts of the coronavirus on the tourism industry. Besides, the study describes the paths that are helpful for academic, researchers, and stakeholders to understand better, respond or behave in the phenomena of the global crisis and reshape, form, and reset the next new normal successful business models in the advent of the pandemic and the era of post-COVID-19. The study discusses responses to the call for the opportunities of transformational research, which develops rationales based on critical discussions that tourism and leisure industry research should conduct further investigations to replicate and reconfirm the general knowledge within the contexts of a pandemic. Instead, the COVID-19 research ought to seek new patterns and pursue novel ways to share information and guidance for tourism futures. The study aims to offer practical, real-time, and theoretical implications on conducting future research in managing, understanding, and transformative valorize the ongoing COVID-19 tourism effects.

This study provides valuable practical insights and helpful implications amid the challenging situation of the COVID-19 crisis. The study offers practical suggestions to manage the employees coping behavior and mental stress. Managers and supervisors employed with tourism and hospitality organizations should focus on employees’ emotions, fear, and anxiety to take safety measures to prevent negative emotions and health behavior at the workplace. The managers of tourism firms should provide employees with clear safety instructions about the transmission of the COVID-19 virus that can help decrease the fear of the virus transmission. The supervisors and management must control employees’ emotions, psychological stress, and anxiety of the COVID-19 spread through various learning programs, social support, medical insurance plans, and skill training (Sheldon and King, 2001). The tourism industry companies should maintain a comfortable and secure working environment as employees with social security and positive emotional state would facilitate the development of useful skills and available resources for firms business growth. Tourism firms employees satisfaction and level of comfort helps achieve better operational and firm performance that improves business growth (Mafini and Pooe David, 2013; Tengilimoglu et al., 2021; Zhang et al., 2021).

The study findings deliver helpful and practical suggestions for economic recovery and tourism revival by stimulating stimulus-response and preventive measures during and after the pandemic of COVID-19. In light of the social costs, relief packages in combating the pandemic should be designed to help the society at a large scale in tourist cities, regions, and destinations which have suffered significantly and experienced adverse socio-economic impacts because of the ongoing pandemic. Conformist policy measures might not be able to manage and overcome the global economic crisis, as this disaster has intensely changed public perception of the risks related to public health concerns associated with travel and tourism. The evidence from China has shown that the tourism industry has initiated partially to regain the momentum in leisure destinations closer to tourists’ homes. Globally, during the weekends, some people have started traveling to closer destinations, but it is still not applicable to main cities at large. The policymakers and decision-makers should formulate recovery strategies with an innovative and holistic mindset rather than just focusing on narrow and direct travel and tourism recovery strategies that were the earlier approaches adopted by many destinations organizing bodies after MERS and SARS. Yang et al. (2020) debated that governments should formulate welfare policies and economic relief packages to respond to adverse consequences of the COVID-19 pandemic. The decision-makers should design packages by allocating financial support across all business sectors, particularly the health, travel, and leisure industry, to ensure a balanced recovery of the affected cities, regions, and residents’ destinations.

This article has critically analyzed the prevailing phenomena of the COVID-19 era and specified some limitations, like any behavioral sciences studies. Nonetheless, this present study has not detracted broader standing to manage and implement protective measures, such as monitoring and executing preventive strategies that help reduce the quick transmission of the infectious disease. Thus, the study’s limitations’ lie in the lack of experimental or clinical exploration. The results related to the coronavirus pandemic are unpredictable. Additionally, future tourism research studies can incorporate empirical data using a cross-sectional research design to add various swaying elements to explore tourists’ mental health wellbeing. This current research findings provide contributions to scientific knowledge with a broader discourse aiming at tourists’ fears and psychological disorders associated with the pandemic. The COVID-19 pandemic is still leading to psychological issues in the masses under the prevailing global health crisis. This research has not created, delivered, or proposed probable new interventional practical strategies and public health policies that minimize tourists’ mental health toll during and after the COVID-19 tourism impacts on the global health crisis and beyond. Hence, upcoming researches should assume the responsibility to incorporate more accurate predictive research models and methods. Surveys about tourists and organizers of the tourism destinations related to post COVID-19 pandemic impacts and travel willingness and leisure products consumption preferences would help practitioners, the academic world, policymakers, and decision-makers foresee the rehabilitated services delivery ecosystem.

There are possibilities of deterioration in the performance of business firms; however, the travel and tourism industry has faced product market competition in the turbulent environment amid the COVID-19 crisis (Ge et al., 2021; Liu et al., 2021; Mubeen et al., 2021a,b). The hotels providing quarantine stations service have experienced a significant decline. More demands for hospital and healthcare center extensions and more dormitories for medical crew to handle the pandemic burden are required. The emergence of the COVID-19 outbreak has resulted in a steep decline in travel and tourism as tourists have had a negative association with the destinations due to their unpleasant experiences about the pandemic horror, suffering, shock, and death stories. The leisure industry’s performance can boost when corporate social responsibility practices are implemented in the industry during and after the global crisis times. Researchers, the academic world, and practitioners can assume the responsibility to conduct longitudinal studies to examine the leisure industry’s performance and explore whether travel and tourism have improved through better innovative marketing strategies. The increasing cases and infection of the coronavirus posed various psychological challenges, fear and anxiety among tourists as well as employees of the hospitality and tourism industry that cuased negative impacts on employees performance. The future research may empirically explore technology adoption impacts on the experiences of the tourists, customers, and destination residents’ participation, loyalty, satisfaction, and hotel brands performance during and after the post-COVID-19 pandemic era. Besides, adopting technology innovation can produce beneficial results to explore the live streaming advantages. Future studies can also explore the post-pandemic impacts and strategies of hotel mergers, acquisitions, and franchise deals to reduce the adverse effects of COVID-19 on tourism. It can help revive the economic activities to bring the next normal to boost economic growth.

## Data Availability Statement

The original contributions presented in the study are included in the article/supplementary material, further inquiries can be directed to the corresponding author/s.

## Author Contributions

All authors listed have made a substantial, direct, and intellectual contribution to the work, and approved it for publication.

## Conflict of Interest

The authors declare that the research was conducted in the absence of any commercial or financial relationships that could be construed as a potential conflict of interest.

## Publisher’s Note

All claims expressed in this article are solely those of the authors and do not necessarily represent those of their affiliated organizations, or those of the publisher, the editors and the reviewers. Any product that may be evaluated in this article, or claim that may be made by its manufacturer, is not guaranteed or endorsed by the publisher.
